# Challenges and opportunities in pragmatic implementation of a holistic hospital care model in Singapore: A mixed-method case study

**DOI:** 10.1371/journal.pone.0245650

**Published:** 2021-01-20

**Authors:** Yi Feng Lai, Sophia Yi-Fei Lee, Jun Xiong, Si Yun Leow, Cher Wee Lim, Biauw Chi Ong

**Affiliations:** 1 MOH Office for Healthcare Transformation, Ministry of Health, Singapore City, Singapore; 2 School of Public Health, University of Illinois at Chicago, Chicago, Illinois, United States of America; 3 Department of Pharmacy, National University of Singapore, Singapore City, Singapore; 4 Business School, National University of Singapore, Singapore City, Singapore; 5 Saw Swee Hock School of Public Health, National University of Singapore, Singapore City, Singapore; 6 Medical Board, Sengkang General Hospital, Singapore City, Singapore; Institute of Mental Health, SINGAPORE

## Abstract

**Introduction:**

Hospital-based practices today remain predominantly disease-oriented, focusing on individual clinical specialties with less visibility on a comprehensive picture of each patient’s health needs. To tackle the challenge of growing multimorbidity worldwide, practices without disease-specific focus have shown better integration of services. However, as we move away from the familiar disease-specific approaches of care delivery, many of us are still learning how to implement generalist care in a cost-effective manner.

**Methods:**

This mixed-method case study, which centred on a specialist-led General Medicine model implemented at an acute hospital in Singapore, aimed to (1) quantitatively summarise its clinical outcomes, and (2) qualitatively describe the challenges and lessons gathered from the pragmatic implementation of the care model. Quantitative hospital data were extracted from databases and summarised. Qualitative staff-reported experiences and insights were gathered through semi-structured interviews and analysed using thematic analysis.

**Results:**

Quantitative findings revealed that the generalist care model was implemented with high fidelity, where more than 75% of patients admitted were placed under General Medicine’s or General Surgery’s care. The mean length of stay was 2.6 days, and the 30-day post-discharge readmission rate was 15%. Inpatient mortality rate was found to be 2.8%, and the average gross hospitalisation bill amounted to SGD3,085.30. For qualitative findings, themes concerning feasibility and operational aspects of the implementation were grouped into categories- (1) Feasibility of ‘One Care Team’ approach, (2) Enablers required for meaningful generalist care, (3) Challenges surrounding information sharing, (4) Lack of integration with the community to facilitate care transition, and (5) Evolving roles of self-management. The findings were rich, with some being identified as barriers that could benefit from system-level de-constraining.

**Discussion:**

This case study was an illustration of our pursuit for an integrated solution to rising prevalence of multimorbidity. While quantitative findings indicated that a pivot towards General Medicine might be possible, data also revealed gaps in clinical outcomes, especially in readmission rates. These findings corroborated with much of the lessons and challenges gathered from qualitative interviews, specifically surrounding the lack of receptacles in the community to facilitate care transition, training, and competency of generalists in holistic management of complex multimorbid cases, as well as inadequate infrastructure to allow information sharing between providers. Thus, a multi-pronged approach might be required to develop a new and sustainable care model for patients with multimorbidity in the long run. In the short to medium transitional period, nonetheless, the specialist-led General Medicine care model demonstrated might be a viable interim approach, especially in circumstances where trained medical generalists remained limited.

## Introduction

Today, with longer life expectancies and shifting lifestyle patterns, populations are expected to have higher prevalence of multimorbidity; herein, multimorbidity is generally defined as the presence of multiple diseases or conditions within the same patient [1, 2]. Such multimorbidity is common and has been rising in prevalence over recent years [3]. In Scotland, for instance, a previous study found that more than 40% of the population (all ages included) had at least one long-term condition and almost 25% of the entire population had more than one long-term condition [4]. However, current hospital-based practices remain predominantly disease-oriented, with less visibility on a comprehensive picture of each patient’s health needs [5, 6]. This eventually contributes to fragmented care, where dynamic care needs are not matched to and addressed by the most appropriate care intervention [7].

International efforts have sought to develop and implement care models centring on the concepts of care integration and holistic care delivery [8–12]. In Europe, such efforts led to the creation of the Joint Action on Chronic Diseases (JA-CHRODIS), emphasising on multi-disciplinary teams constantly reviewing care plans for patients, customising self-management tools and sharing information across different platforms and settings [13]. In Canada, as part of the PRISMA care model, case managers were assigned to helm and coordinate patients’ care needs between care teams in the community [14].

In Singapore, the government’s restructuring of public healthcare institutions from a model that was publicly-owned to one that is publicly-funded but privately-run back in 1980s provided the impetus for these institutions to adopt a more competitive posture towards care delivery [15, 16]. As the population was younger with less chronic disease needs, medical specialisation was encouraged during the period of rapid advances in medical sciences that saw the establishment of six national specialty centres [17, 18]. It contributed, among other factors, to the development of single-disease-oriented approaches to care delivery. In addition, public hospitals today account for 8 in 10 of acute hospital beds and acute inpatient admissions in Singapore, while 20 public polyclinics, representing 20% of the country’s primary care capacity, manage about 40% of attendances for non-communicable diseases [19–22]. This disproportionate utilisation of public sector resources for tertiary acute care and complex primary care, coupled with changing patients’ needs, exerts growing pressure on the public healthcare system and may further exacerbate the fragmentation of care in the long run [16, 23].

Patients with acute issues, especially those who exhibit unclear presentations or present with multiple medical conditions, are best managed during their hospital stay by a dedicated care team with broad-based clinical capabilities [13, 14]. Herein, timely, holistic and responsive management can help to reduce potential fragmentation of care, and allow a broad-based scope of resources to most appropriately attend to the complexity of care needs.

As we move away from the familiar disease-specific approaches of care delivery, many of us are still learning how to implement generalist care in a cost-effective manner. Through closely examining a pragmatic implementation of a generalist care model in Singapore, this study attempted to examine the safety, efficacy, challenges and lessons pertaining to such care model’s roles at a regional acute hospital and its community partners in Singapore. The findings could also reveal issues and insights that were perhaps not as well anticipated or articulated during the hospital’s initial planning and conceptualisation of its care model, which might become useful lessons for other hospital clinicians and administrators attempting similar care redesign efforts in the future.

## Method

A mixed-method case study was conducted at Sengkang General Hospital (SKH), a 1000-bed acute hospital in the north-eastern region of Singapore that opened in July 2018. SKH was part of an integrated hospital campus alongside a 400-bed Sengkang Community Hospital (SKCH). The co-location of SKH and SKCH facilitated a multi-disciplinary approach and a seamless transition of care across both institutions.

### Setting

The hospital’s General Medicine care model built its foundation on a generalist approach to multimorbidity. It was implemented with a team of specialist physicians practising General Medicine. They consisted of doctors who were trained in family medicine, internal medicine, geriatric medicine, rehabilitation medicine and a wide array of other specialties. With defined General Medicine protocols, the model predicated on team-based integration of specialist expertise, which shifted organ-based diagnosis and treatment to interactive, system-wide intervention.

A “round-robin” team-based approach saw the General Medicine teams managing assigned patients at various ward locations. The consultants, regardless of their trained specialty, were required to devote a significant portion of their time practising General Medicine, and caring for patients with conditions that often did not fall within their respective trained specialty. When required, referrals could be made to request for other specialists’ input for management of complex issues requiring advanced tertiary care. Proper discharge planning supported by a team of patient navigators (case managers) was also a purposefully-included feature of the care model. Besides, the General Medicine team also provided support to selected surgical patients in managing the chronic diseases pre- and post-surgeries. With a healthcare ecosystem approach, designated General Medicine physicians conducted post-discharge house visits to patients at risk of frequent readmissions (Hospital-to-Home Programme) during the study period, and provided medical support to institutionalised patients in the community, such as those at Institute of Mental Health, destitute and nursing homes within the precinct through a “hub and spoke” approach.

### Data collection and analysis plan

To evaluate the clinical efficacy of its service implementation, de-identified quantitative demographic and clinical data including length of stay, 30-day readmissions, inpatient mortality and gross hospitalisation bills were extracted from hospital information system for General Medicine patients admitted between October 2018 and March 2019. Extraction and de-identification of data was exclusively performed by a hospital-authorised staff not involved in the conduct of this study. Demographic data were presented using measures of central tendency and proportions. As length of stay and gross hospitalisation bill data had skewed distributions, geometric mean and 95% confidence intervals (CI) were used to summarise the variables. Readmissions and inpatient mortality were presented as proportions.

For staff-reported outcomes, experiences and insights, semi-structured individual or group interviews were conducted in SKH by study investigators until data saturation over a period of 6 weeks from April 2019. For the development of the interview topic guide, three domains of interests were considered: (1) understanding SKH’s care model, (2) describing perceptions on the current state of care model implementation and its challenges, (3) identifying lessons from implementation and future opportunities.

The interview participants were key stakeholders responsible for the planning, implementation and scaling of care model. These participants included the senior leaders as well as frontline staff members. All identified staff were individually approached via e-mail and voluntarily recruited from a wide variety of departments, including medical, nursing, allied health, administration, clinical governance, and strategic planning and management. Before the interview, the study objectives and rights of participation were explained to all participants. Permission to audio-record the interview was also sought. Their identities have been protected using pseudonyms. All interviews were conducted in English.

Thematic analysis was used to identify and analyse patterns from the qualitative data through a pre-determined conceptual framework (Table 1).

The framework took reference from a locally-developed Integrated General Hospital care model, Canada’s PRISMA model and European-led Joint Action on Chronic Diseases’ care framework [13, 14, 24]. Aspects and elements of the care model were categorised into (1) Multidisciplinary single care team, (2) Care enablers, (3) Data sharing, (4) Community Integration, and (5) Patient empowerment.

**Table 1:** Comparison of themes among international models.

	Multi-disciplinary Single Care Team	Care Enablers	Data Sharing	Community Integration	Patient Empowerment
**JA-Chrodis (Europe)**	**Delivery System design** • Regular Comprehensive assessment • Multi disciplinary team • Individualised Care Plan • Appointment of case manager	**Decision Support** • Implementation of evidence-based medicine • Team training • Developing consultation system to consult professional experts outside of core team	**Clinical Info System** • Electronic patient records and computerised clinical charts • Exchange of patients’ information • Uniform coding of patients’ health problems • Patient platforms allowing patients to exchange information with their care providers	**Community Resources** • Access to community resources • Involvement of social network • Psychosocial support	**Self-Management Support** • Training of care providers to tailor self-management support for patients • Providing options for patients to improve their health literacy • Patient education • Involving family members and family education • Offering approaches to strengthen patients’ self-management and self-efficacy • Involving patients in decision-making • Training patients to use medical devices, supportive aids and health monitoring tools correctly
**PRISMA (Canada)**	• Coordination between institutions, managers, and decision-makers • Individualised service plan • Case Manager	• Single point of entry • Single assessment instrument	• Information system		
**Integrated General Hospital (Singapore)**	• Ensure early identification of needs & interventions, beyond medical domains • Assign one care team per patient, led by an attending doctor with specialist reinforcement.	• Deliver benchmarked care assessment • Provide open adaptive infrastructure, that supports iterative care redesign and technology insertion • Staff manpower dynamically, according to needs • Develop and maintain one shared care plan across providers	• Provide one shared platform for needs matching and referral management	• Track longitudinal outcomes	• Training patients and caregivers for post-discharge care • Provide resource registries for self-management

Following verbatim transcription of the interviews, open coding was done independently by a core team of three study team members. This required them to summarise the participants’ responses into codes. Similar patterns of behaviour or accounts were stored in ‘nodes’, which were developed within and across transcripts. These were then categorised and recorded as themes and sub-themes within the conceptual framework. Disagreements were reconciled through group discussion and consensus. Qualitative data management was supported by ATLAS.TI version 8.

Pursuant to the prevailing Human Biomedical Research Act 2015 in Singapore, this study fell under the category of service evaluation and clinical audit, and thus did not require specific ethics approval. Administratively, the evaluation protocol, data acquisition plan, and the proposal to publish aggregated data were reviewed and approved by the hospital’s medical board and senior management before data collection commenced.

## Results

Hospitalisation data from 1 Oct 2018 to 31 March 2019 at SKH was analysed to obtain the patient profile. Key demographic variables were summarised in Table 2.

**Table 2:** Demographic characteristics of patients cared for at SKH inpatient wards.

Variables	Observations (n = 17148)
**Age, mean year (SD)**	60.2 (19.5)
**Male sex, %**	51.2
**Ethnicity, %**	
Chinese	69.2
Malay	14.2
Indian	9.8
Others	6.8
**Financial Class, %**	
A (No subsidy from Government)	4.7
B1	5.5
B2	19.1
C (Most subsidy from Government)	62.9
**Admission from ED, %**	88.9
**Admission Specialty, %**	
General Medicine	59.5
General Surgery	16.9
Subspecialties	23.6
**Charlson Comorbidity Index (CCI), %**	
0	65.7
1–5	23.4
≥6	10.9
**Residing within catchment area, % (95% CI)**	
No readmission	71.0 (70.3–71.7)
At least 1 episode	68.8 (66.8–70.9)

Abbreviations: ED, Emergency Department; CCI, Charlson Comorbidity Index; SD, standard deviation; CI, confidence interval.

At SKH, the patients receiving General Medicine care had an average length of stay (ALOS) of 2.6 days, with 15% of 30-day readmission rate, 2.8% of inpatient mortality and incurred an average of $3,085.30 for their hospitalisation episodes (Table 3).

**Table 3:** Summary of quantitative findings from SKH implementation (Oct 2018-Mar 2019).

	SKH, n = 9981
Length of stay, day (95% CI)^+^	2.6 (2.6, 2.7)
Readmission, %	15
Inpatient mortality, %	2.8
Total hospitalisation cost, $ (95% CI)^+^	3,085.3 (3,038.3, 3,133.0)

Abbreviations: CI, confidence interval.

+Geometric mean (95% CI).

For qualitative data collection, a total of 20 interviews were carried out among 24 individuals (Table 4). Approximately half of them (n = 13) did not have patient-fronting job functions and most of them (n = 18) held managerial or leadership appointments. While hospital managers and leaders were able to articulate vision and intention of the implemented care model, many frontline staff spoke at length about their perceived challenges and opportunities arising from SKH’s implementation of the General Medicine care model.

**Table 4:** Summary of interview participants’ characteristics.

ID	Department	Job Function	Seniority	Interview Duration
SM01	Family Medicine	Clinician	Management	1:24:20
SM02	Family Medicine	Clinician	Frontline	1:17:57
SM03	General Medicine	Clinician	Management	1:18:50
SM04	Family Medicine	Clinician	Management	1:12:49
SM05	Orthopaedic Surgery	Clinician	Management	2:00:35
SF06	Allied Health	Administrator	Senior Management	1:08:25
SM07	Strategic Planning and Management	Administrator	Senior Management	59:58
SM08	Nursing	Clinician	Frontline	1:06:09
SF09	Nursing	Administrator	Senior Management	1:09:47
SF10	Medical Board	Administrator	Senior Management	38:41
SM11	Family Medicine	Clinician	Frontline	1:11:33
SM12	Strategic Planning and Management	Administrator	Management	1:18:00
SM13	Family Medicine	Administrator	Senior Management	1:11:32
SF14	Strategic Planning and Management	Administrator	Management	1:16:24
SF15	Clinical Governance	Administrator	Management	1:04:06
SFFF16	Nursing	Clinician	Frontline	1:12:28
SFFF16	Nursing	Clinician	Frontline	
SFFF16	Nursing	Administrator	Management	
SFFF17	Physiotherapy	Clinician	Frontline	1:34:54
SFFF17	Occupational Therapy	Administrator	Management	
SFFF17	Medical Social Service	Administrator	Management	
SF18	Emergency Medicine	Clinician	Management	1:16:23
SF19	Strategic Planning and Management	Administrator	Management	49:46
SF20	Strategic Projects	Administrator	Management	47:19

### Multidisciplinary single care team

Instead of solely relying on traditional generalists with background in internal medicine (IM), family medicine (FM) and geriatric medicine (GRM), SKH enlisted other specialists to practise General Medicine on top of existing specialist duties (e.g. seeing referred subspecialty cases, running specialist outpatient clinics). While this “specialist-led General Medicine” approach drew initial concerns, most clinicians have since embraced it; some clinicians even reported how they derived satisfaction from treating multimorbid patients akin to “*solving a complex jigsaw puzzle*”.

Notwithstanding, the reality of establishing a single specialist-led General Medicine care team in an inpatient setting might introduce unanticipated issues.

#### Unclear designation of roles and responsibilities

Participants shared that, while the role of the lead specialist was clear in coordinating inpatient care, there remained concerns on how care and care planning could best be shared with each patient’s primary care physician.

…who is going to be the driver of it, you see. Who’s going to do it? Is it going to be the (hospital) generalist, the family physician…? **SM01**

To this, some suggested implementation of family medicine (FM) hospitalist model, where family physicians trained in General Medicine could also practise in an inpatient setting and eventually take on the role of principal doctor upon a patient’s hospitalisation, given their wider knowledge on available community resources and ability to continue care beyond discharge [25, 26]. Some also suggested working towards identifying and transferring care to named family physicians in the community as the long term care coordinators for patients with multimorbidity.

#### Lack of coordination when making care plans

Some participants raised the issue of a lack of intra-hospital coordination and communication among doctors and nurses when making care plans. This could be attributed to difficulty in coordinating schedules among multiple physicians, and an inherent lack of incentives and performance measurements for proactive patient sharing.

I need to hit my KPI (Key Performance Indicators)…to let my CMB (Chairman, Medical Board) see the number of patients I see, so why do I want to share my patients? So in the end, it's number… because you are (being evaluated) based on the KPI … **SFFF16**

Furthermore, territorial practices among different specialties may also potentially hamper care collaboration.

### Care enablers

#### Patient characterisation

A few participants raised concerns over the need for benchmarked patient segmentation framework(s) during inpatient admission and referrals. Patients’ medical conditions and psychosocial profiles were at times seen to be subjectively assessed without standardised definitions and criteria.

now it’s (patient assessment) not very standardised…what the patient gets referred to depends a lot on the individual who’s doing the assessment…based on their expertise and their knowledge… SF18

Participants also touched on the merits and implementation difficulties in establishing an objective standard to classify patients into different levels of acuity, especially for patients with sub-acute conditions.

To achieve clearer segmentation of patients, some opportunities were suggested, including implementing longitudinal risk stratification, identifying patients’ activation level and readmission risks, and using customised pathways to provide more targeted interventions for specific patient archetypes.

### Data sharing

#### Barriers of access to patient’s information

One of the most frequently raised challenges pointed at the lack of a common system to easily retrieve information on patients’ key needs and care journey across different providers. This was often coupled with concerns over restrictive access to their information, in part due to regulations like Personal Data Protection Act (PDPA).

nursing home partners do have their own clinical records system, it is not integrated with the hospitals clinical record system, so when patients are being discharged… some of these information flow through is not there **SM12**

#### Lack of efficient communication for information sharing

Another notable challenge lied in the communication of information between hospital doctors across different healthcare clusters. Information shared might sometimes be incomplete or unstructured.

### Community integration

#### Resource availability

Apart from limited intra-hospital resources, participants raised concerns on resource availability and a hope for more support services among community providers. There was concern that General Practitioners (GPs) and Polyclinics might not be sufficiently equipped with diagnostic resources. Furthermore, they lacked access to support services for investigative and specialised tests, which could limit the acuity and complexity of multimorbid cases that they could manage.

Participants suggested improvements for more efficient patient journeys, such as privileging primary care partners the rights to order more advanced diagnostic tests and to allow direct referrals to allied health and nursing resources. Suggestions were also made to leverage on dynamic resource concepts like swing beds and virtual wards in the community.

#### Competency of community providers

Another concern surfaced was the perceived lack of competency among community care providers. A number of participants echoed the sentiment that primary care providers were not ready to accept patients discharged from the hospital, ostensibly due to skill mix mismatch. A specific concern was in the perceived variable quality in care that GPs provided.

And those GPs who are not well trained, they also struggle. They feel that they are not adequately trained to deal with chronic diseases, so they keep referring to polyclinic or worse, back to specialist. **SM04**

Nonetheless, the participants also acknowledged systemic limitations that community partners faced. For instance, GPs might lack the exposure and experience in handling cases with medical and/or social complexity. This might result in a lack of confidence among GPs to manage cases requiring post-discharge transitional care.

If… the GPs don’t see these cases, they get **rusty**, and patients don’t have confidence, and all the more patients don’t want to go there. **SM05**

#### Absence of family/social support

Participants also brought up the challenge of a lack of family and social support in the community network. Particularly, the more vulnerable patients who were less mobile, more ‘socially isolated’, or had little motivation to adhere to prescribed treatment plans. This increased their risk of being “lost to the system” and subsequently developing exacerbated medical conditions.

### Patient empowerment

A patient’s knowledge, skills and confidence towards self-management played an important role in his/her empowerment. One challenge surfaced was the high readmission rates through ED, as many of such patients were observed to lack basic self-care knowledge or motivation to self-administer medications or treatments.

There are people who, for various reasons, really truly lack of support, no caregivers you know. I mean there will also be subsets who are like…poor health literacy…don’t understand that there is a need to have regular follow-up. **SF02**

Furthermore, there were distorted expectations of hospital services, where patients could become over-reliant on hospital services and less inclined to seek care within the community. This over-reliance could eventually put a strain on the limited hospital resources.

*What do they expect from the hospital*? *With the way the feedback and complaints are coming*, *it’s not just medical anymore*. *It's everything else like a hotel*. *Hospitals are not built for that*. **SF18**

To better empower patients to take care of their health, and encourage family members and caregivers to participate and support, it was suggested for the hospital to adopt a more patient-centric perspective, and to collaborate with relevant parties like Health Promotion Board to organise health screening activities, health education and self-care coaching sessions.

## Discussion

SKH’s implementation of a holistic hospital care model could inform further discussions on multidisciplinary single care team, care enablers, data sharing, community integration and patient empowerment.

These endeavours pointed to opportunities that an integrated care model could be an effective enabler in delivering more holistic and coordinated care, including but not limited to the hospital setting.

The care model supported the notion that a General Medicine focused approach could provide appropriate holistic inpatient care, especially for a growing population of patients with multimorbidity. Overall, SKH achieved a high direct admission rate to General Medicine at almost 60%, with vast majority of patients discharged without any transfer of lead physician during their inpatient stay. The rate of direct admission to General Medicine was significantly higher compared to other acute hospitals in Singapore, achieving between 20–50% of total admissions [27]. While shorter average length of stay was observed (2.6 days vs 5.7 days) compared to published data from another local hospital’s specialists-led care model, readmission rates appeared elevated (15% vs 7.5%) [28]. Inflation-adjusted hospitalisation bill sizes were similar at both hospitals (SGD3,085.30 vs SGD3,123.60) while inpatient mortality was significantly lower (2.8% vs 5.3%) [29]. These results further attested the notion that the generalist model might not only be able to produce comparable results when compared to specialists-led care, but was also suitable for patients across a wide range of acuity level. This also corroborated with qualitative accounts, and alluded to opportunities to extend a similar multidisciplinary single team care approach beyond the hospitalisation episode. For instance, playing the role as the primary care provider or coordinator, lead physicians and case managers should perhaps not only coordinate clinical inputs across professional domains, but also be equipped with trans-professional acumen and patient engagement tools to anticipate, navigate and manage post-discharge care complexities.

SKH’s implementation showed that hospital inpatient care remained accessible to most patients and might involve low barriers of entry. For instance, about 89% of SKH’s patients were admitted through Emergency Department. This was facilitated by the hospital’s strategic proximity to key neighbourhoods, which resulted in approximately 70% of SKH’s patients coming from the immediate precinct around the hospital. This presented new opportunities for interventions to encourage service integration with community providers [30–32].

From the qualitative interviews, three improvement opportunities were identified.

Firstly, for existing physicians practising General Medicine, a clearer training roadmap might be beneficial to help guide them to acquire the necessary skillset and experience to perform their medical duties. General Medicine should continue to innovate and reinvent itself in order to deal with increasingly complex patients with multiple comorbidities. Retaining medical specialties in a consultative role with inpatient medical care managed by a new model of future General Medicine hospitalists could improve efficiency while maintaining the quality of care. Training reforms might be needed to address the training-practice gap in General Medicine by extending postgraduate training to provide sufficient opportunities for mastery of core skills [33]. In addition, an outcomes-driven training process would also be useful moving forward [34]. However, efforts to reform medical education must be done with deliberate attention to revisit the training content to suit the future practice models, address issues including service obligation, and experiment the best possible solutions.

Secondly, better patient characterisation, segmentation, and information flow would help clinicians and administrators allocate resources to the patients more appropriately. At the same time, other non-medical information including social needs, functional status and activation level might be useful and should be captured before discharge to facilitate shared care planning with community providers.

Finally, collaboration between stakeholders, especially in discharge planning could be further strengthened. Healthcare leaders should continue to emphasise on building teamwork and collaboration between generalists and specialists in the crafting of the patients’ care plans. Clinical pathways should also be designed and structured in ways that would prompt and facilitate deliberate and purposeful discharge planning. The use of patient navigators or care coordinators should also be mainstreamed to allow initiation and timely follow-up of discharge planning conversations, especially for patients who might require transitional care.

While many interview participants welcomed SKH’s holistic approach, it would be useful to also consider key challenges highlighted in delivering General Medicine primarily at the acute hospital, such as patient ownership post-acute care. Many patients had care needs that appeared to be increasingly sandwiched between acute and post-acute care. Operationally, it remained challenging to definitively describe and prescribe the point of transition from acute care to post-acute care. Additional care redesign could be considered in appreciating and finessing SKH’s model further. This could perhaps include identifying more resource-efficient care settings–those anchored in the community, for instance–for satellite General Medicine practices.

Paradoxically, with a strong system of specialist care in Singapore, General Medicine care is becoming increasingly important in the healthcare landscape today. While many acute hospitals might have succeeded in delivering high quality specialist care at affordable subsidised prices, an unintended consequence of that was an induced willingness among patients to get admitted into hospital to see multiple specialists for multiple conditions instead of seeing a good generalist for holistic care. Such mindset needs to be corrected so that right-siting of care to the right care setting with the corresponding amount of resource utilisation, especially healthcare manpower, can be achieved. This further underscores the importance of strengthening the existing generalist care model to better tackle immediate healthcare challenges in many mature healthcare systems caring for multimorbid, ageing populations.

## Conclusion

The findings from SKH’s implementation highlighted the importance for continuing efforts to better understand the implementation contexts for hospital care redesign and to describe successful local approaches to holistic care delivery. This can inform how medical inpatient services can be best structured and enabled in delivering productive and coordinated hospital care to a growing patient population with multiple care needs.

In the short to medium transitional period, however, SKH’s care model may be emulated, especially in circumstances where supply of trained medical generalists remain inadequate [35]. In the longer term, opportunities for care redesign lie in talent development, systematic evaluation, focused system-level data analytics, financing reform and technology enablement. With more regular exchanges on care redesign approaches between key stakeholders, the system can be more ready to converge towards a more sustainable and adaptive framework that better caters to emerging care needs.

## Supporting information

S1 File (DOCX)

## References

[1] Federal Health Gazette—Health Research—Health Protection, 2011, Volume 54, Number 8, Page 907.

[2] Doblhammer G., Kreft D., Uijen AA, Van de Lisdon EH. Multimorbidity in primary care: prevalence and trend over the last 20 years. Eur J Gen Pract 2008;14:28–32. 10.1080/13814780802436093 18949641

[3] Uijen AA, van de Lisdonk EH. Multimorbidity in primary care: prevalence and trend over the last 20 years. Eur J Gen Pract. 2008;14(s1):28–32. 10.1080/13814780802436093 18949641

[4] Barnett K, Mercer SW, Norbury M, Watt G, Wyke S, Guthrie B. Epidemiology of multimorbidity and implications for health care, research, and medical education: a cross-sectional study. Lancet. 2012;380(9836):37–43. 10.1016/S0140-6736(12)60240-2 22579043

[5] Palmer Katie, Marengoni Alessandra, Maria João Forjaz, et al Multimorbidity care model: Recommendations from the consensus meeting of the Joint Action on Chronic Diseases and Promoting Healthy Ageing across the Life Cycle (JA-CHRODIS), Health Policy, Volume 122, Issue 1, 2018, Pages 4–11, ISSN 0168-8510, 10.1016/j.healthpol.2017.09.006 28967492

[6] Rijiken Mieke, et al Managing multimorbidity: Profiles of integrated care approaches targeting people with multiple chronic conditions in Europe☆- Health Policy, 1 2018, Volume 122, Issue 1, Pages 44–52. 10.1016/j.healthpol.2017.10.002 29102089

[7] Rijken M, Hujala A, van Ginneken E, Melchiorre MG, Groenewegen P, Schellevis F. Managing multimorbidity: Profiles of integrated care approaches targeting people with multiple chronic conditions in Europe. Health Policy. 2018;122(1):44–52. 10.1016/j.healthpol.2017.10.002 29102089

[8] Connor M, Cooper H, McMurray A. The Gold Coast Integrated Care Model. International Journal of Integrated Care. 2016;16(3):2 10.5334/ijic.2233 28435415 PMC5350640

[9] Curry N, Ham C. Clinical and service integration The route to improved outcomes. The King’s Fund; 2010 Available from https://www.kingsfund.org.uk/sites/files/kf/Clinical-and-serviceintegration-Natasha-Curry-Chris-Ham-22-November-2010.pdf. Accessed on 4 Sep 2019.

[10] Ham C, Glasby J, Parker H, Smith J. Altogether now? Policy options for integrating care. Health Services Management Centre. 2008 Available from http://www.wales.nhs.uk/sitesplus/documents/829/HSMC%20Report%20-%20Altogether_Now_Report.pdf. Accessed on 4 Sep 2019.

[11] Perlin J, Kolodner R, Roswell R. The Veterans Health Administration: Quality, Value, Accountability, and Information as Transforming Strategies for Patient-Centered Care. Am J Manag Care. 2004 Available from http://www.ajmc.com/journals/issue/2004/2004-11-vol10-n11Pt2/Nov04-1955p828-836/. Accessed on 4 Sep 2019. 15609736

[12] Suan Ee Ong Shilpa Tyagi, Jane Mingjie Lim Kee Seng Chia, Helena Legido-Quigley, Health systems reforms in Singapore: A qualitative study of key stakeholders, Health Policy, Volume 122, Issue 4, 2018, Pages 431–443, ISSN 0168-8510, 10.1016/j.healthpol.2018.02.005 29478876

[13] Onder G., Palmer K., Navickas R., Jureviciene E., Mammarella F., Strandzheva M., et al Joint Action on Chronic D, Promoting Healthy Ageing across the Life C. Time to face the challenge of multimorbidity. A European perspective from the joint action on chronic diseases and promoting healthy ageing across the life cycle (JA-CHRODIS) European Journal of Internal Medicine, 26 (2015), pp. 157–159. 10.1016/j.ejim.2015.02.020 25797840

[14] Hebert Rejean, J Durand Pierre, Dubuc Nicole & Tourigny André (2003). PRISMA: a new model of Integrated Service Delivery for the Frail Older People in Canada. International journal of integrated care. 3 e08 10.5334/ijic.73 16896376 PMC1483944

[15] Ramesh, M. 2017 Singapore’s healthcare success did not come cheap. Available from https://www.channelnewsasia.com/news/singapore/commentary-singapore-s-healthcare-success-did-not-come-cheap-or-8876004. Accessed on 4 Sep 2019.

[16] Nurjono M, et al Implementation of Integrated Care in Singapore: A Complex Adaptive System Perspective. International Journal of Integrated Care, 2018; 18(4): 4, 1–7. 10.5334/ijic.4174 30483037 PMC6196576

[17] Ministry of Health Singapore. Healthcare Services and Facilities. Available from https://www.moh.gov.sg/home/our-healthcare-system/healthcare-services-and-facilities. Accessed on 11 Aug 2020.

[18] MOH Holdings. Caring for our people- 50 years of healthcare in Singapore. Available from https://www.mohh.com.sg/Documents/book/caring-for-our-people-50-years-of-healthcare-in-singapore.pdf. Accessed on 11 Aug 2020.

[19] Ong B. The Future of Singapore Healthcare. Medicine; a publication of the Yong Loo Lin School of Medicine, 2016; 20: 20–23.

[20] Fund, TC. 2015 International Profiles of Health Care Systems. In: Mossialos, E, Wenzl, M, Osborn, R and Sarnak, D (eds.); 2015.

[21] Khoo HS, Lim YW and Vrijhoef HJM. Primary healthcare system and practice characteristics in Singapore. Asia Pacific Family Medicine, 2014; 13(1): 8–8. 10.1186/s12930-014-0008-x 25120380 PMC4129466

[22] Ministry of Health Singapore: Primary Care Survey 2010-profile of primary care patients. 2010. Available from https://www.moh.gov.sg/content/moh_web/home/Publications/information_papers/2011/primary_care_survey2010profileofprimarycarepatients.html. Accessed on 4 Sep 2019.

[23] Maruthappu Mahiben, Hasan Ali & Zeltner Thomas (2015) Enablers and Barriers in Implementing Integrated Care, Health Systems & Reform, 1:4, 250–256, 10.1080/23288604.2015.1077301 31519094

[24] Alexandra Hospital pioneers Singapore's Integrated General Hospital care model. Available from: https://www.ah.com.sg/Documents/Alexandra%20Hospital%20pioneers%20Singapore%27s%20Integrated%20General%20Hospital%20care%20model%20-%2014%20December%202018.pdf. Accessed on 10 Nov 2020.

[25] Buch MS, et al Implementing Integrated Care–Lessons from the Odense Integrated Care Trial. International Journal of Integrated Care, 2018; 18(4): 6, 1–9. 10.5334/ijic.4164 30386188 PMC6208289

[26] Society of Hospital Medicine. Mission statement and goals. 1997. Available at: http://www.hospitalmedicine.org/Content/NavigationMenu/AboutSHM/MissionStatementGoals/Mission_Statement_Go.htm.

[27] Ministry of Health Singapore (2019). Direct inpatient admissions to General Medicine. Unpublished manuscript.

[28] Lee KH, Yang Y, Yang KS, Ong BC, Ng HS. Bringing generalists into the hospital: outcomes of a family medicine hospitalist model in Singapore. J Hosp Med. 2011 3; 6: 115–21. 10.1002/jhm.821 21387546

[29] Monetary Authority of Singapore. Goods & Services Inflation Calculator. Available from: https://secure.mas.gov.sg/calculator/goodsandservices.aspx. Accessed 4 Sep 2019.

[30] Swedish Institute. Healthcare in Sweden. Available from https://sweden.se/society/health-care-in-sweden/. Accessed on 13 Aug 2020.

[31] Maass MC, Asikainen P, Mäenpää T, Wanne O, Suominen T. Usefulness of a Regional Health Care Information System in primary care: a case study. Comput Methods Programs Biomed. 2008;91(2):175–181. 10.1016/j.cmpb.2008.04.005 18514363

[32] Hendrickson MA, Obeya E, Wey AR, Gaillard PR. Community Primary Care Provider Preferences for Emergency Department Follow-up Recommendations: A Regional Study. Pediatr Emerg Care. 2017;33(10):690–693. 10.1097/PEC.0000000000001068 28277413 PMC5591753

[33] Larson E.B. Society of General Internal Medicine (SGIM) Task Force on the Domain of General Internal Medicine. Abstract health care system chaos should spur innovation: summary of a report of the Society of General Internal Medicine Task Force on the Domain of General Internal Medicine. Ann Intern Med. 2004; 140: 639–643. 10.7326/0003-4819-140-8-200404200-00011 15096335

[34] Goroll AH, Sirio C, Duffy FD, et al A new model for accreditation of residency programs in internal medicine. Ann Intern Med. 2004; 140: 902–909. 10.7326/0003-4819-140-11-200406010-00012 15172905

[35] The Straits Times- MOH reviews doctors' training to become specialists. Available from: https://www.straitstimes.com/singapore/health/moh-reviews-doctors-training-to-become-specialists. Accessed on 10 Nov 2020.

