# Supplementary material for: Challenges and opportunities in pragmatic implementation of a holistic hospital care model in Singapore: A mixed-method case study
Source: PLoS One. 2021 Jan 20;16(1):e0245650. doi: 10.1371/journal.pone.0245650 (PMC7817047; doi:10.1371/journal.pone.0245650)
Supplement: S1 File — (DOCX) [file pone.0245650.s001.docx]

**Appendix A**

**GENERALIST CARE MODEL ENVIRONMENTAL SCAN**

**PERSPECTIVES AND VIEWS ON HOLISTIC, WHOLE-PERSON CARE MODEL AMONG KEY STAKEHOLDERS IN SENGKANG GENERAL HOSPITAL (SKH)**

Aim:

To explore the experience, perception and opinion of key healthcare leaders regarding the planning, execution and monitoring of Generalist Care Model in Sengkang Health and Sengkang General Hospital.

Specific objectives:

- Primary objective: To understand, describe and aggregate relevant approaches adopted by SKH in delivering timely, Coordinated, Quality Care and driving professional productivity.
- Secondary objectives:
  - To compare and contrast the various Generalist approaches with AH’s practice. This will complement AH’s concurrent evaluation of its IGH implementation, which is expected to finalize over 2020; and
  - To curate and share with hospital providers a portfolio of IGH-relevant ideas, success stories and best practices.

**Semi-structured interview topic guide**

Self-introduction

*Assurance of data confidentiality:*

Issues discussed today and related recordings, transcripts and associated data will be treated confidentially. While participants’ identity cannot be masked during data collection, they will not be revealed when results are collated subsequently. Only aggregated responses and findings will be presented to external parties, which may include the management of MOHT, MOH PRED and Sengkang General Hospital. Interview recordings will be transcribed, coded and analysed using thematic analysis.

Background information:

1) How long have you been working in healthcare and in SKH Generalist Care Model respectively?

2) What were your previous portfolios?

3) Title, profession, specialty, years of practice, home institution, relevant experience (work & non-work)

|  | **Questions** | **Optional probes** |
| --- | --- | --- |
| Understanding of the SKH Care Model | What do you understand by the SKH Care Model?   - *Timely, Coordinated, Quality Care by a Connected Network of Healthcare Partners*   What issues does SKH care model set out to solve?  <refer to Appendix B: Generalist issues>  Apart from the issues you have identified, do you see these other issues as potentially worth solving? | Are you able to share what maybe the key components that best describe SKH care model?  *Integrated Practice Units (IPU)^[[1]](#footnote-1)^, Team-Based Integration of Specialist Expertise for General, Whole-Person Care*  (On a piece of paper) How would you map the issues to the solutions arising from the SKH care model?  For the issues worth solving, how would you approach them in alignment with SKH care model? |

*<Pass Appendix C and Appendix D to participant>*

Appendix B is an extract from the Media Release in December 2018. It describes the 5 key differentiating elements of the Generalist model. Appendix C is a summary of the current SKH Care Model. You may take a minute or two to go through these appendices.

Model specific questions:

|  | **Questions** | **Optional probes** |
| --- | --- | --- |
| Perception on the current state of care model implementation and challenges at SKH | Inpatient care  D/c and care planning upfront  - Parallel with Generalist (d)?  Primary physician for every patient  - Parallel with Generalist (a)?  - Is this practice sustainable? Why?  AMDU to ward-based team mgt  - Parallel with Generalist (b)?  Outpatient care  Right-siting to primary care  - What role do you think SOC/MC should play in the post-discharge care of the patients?  Early specialist care goal  - At what stage is it communicated and how?  Specialist support for primary care  - How do you envision this being done?  Expedited return to SOC  - How do you envision this being done?  Community care post-discharge  - I’m aware that SKH has been working on community outreach. Are you able to share with me any good work that is being carried out with regards to community care post-discharge?  Collaborations  GH-CH transfers  - How do you envision this being done?  Backup:  Challenges  -Manpower  -Operational effectiveness & readiness  -Warehouse supply management  -Hospital facilities & resources  -Research, innovation  -Education  How is the model different from other institutions where you might have come across?  Do you think your colleagues on the ground have sufficient knowledge and understanding towards SKH care model to implement the initiatives? | -What are some of the challenges faced during early d/c planning?  -Did the idea of creating a Department of Gen Med work to encourage generalist behaviour?  - Why the change?  - Are these initiatives equally applied to all admitted patients? If not, what may be the admission criteria?  -What do you think of IGH (c)’s approach of having 1 single SOC appt to Gen Med post-discharge  Challenges/Enabler needed?  Challenges/Enabler needed?  Challenges/Enabler needed?  - Is there any specific plan to enable patients for self-managed care in the community, with or without the help of community service providers?  In realizing minimal transfers, how do you overcome issues wrt subventions?  -Revamping of education/training?  Have you come across any comments made by your colleagues from other institutions towards our model of care?  How sufficient do you think the senior management communicates strategic directions such as care integration to the ground staff? |
| lessons from past implementation and future opportunities | Lessons  - In the course of developing and testing SKH care model, what are some of the lessons you may want to highlight?  - Has the ward design been able to complement the care model?  Parallel with Generalist (e)?  What have been the key facilitators?  Key constraints? | Were the programs and initiatives implemented as envisioned?  How would you have tackled the issues differently if you have the chance to redo?  What have been the experiences of staff delivering it? |
| Future works | Are you happy with our current SKH Model? Do you see a need to change or further enhance it?  Retrospectively, if you are able to suggest other elements to a “novel” integrated, holistic, whole-person care model, what may they be?  Room for Generalist Model  Do you see a need for a different approach to care for patients who need inpatient management but who may not require specialised tertiary care, especially for who have multiple chronic medical conditions?  Optional:  Opportunities  - Data analytics  - Simulation to manage ED manpower  What are some of the further changes and improvement you hope to see?   - Care Redesign - Tech Insertion - Job Redesign - Logic Model Validation - Agile Processes   What are some potential ideas, or even policy enhancements, we may consider? | Have these elements been validated in parts or implemented locally by other institutions?  <refer to Appendix E>  Does this model make sense to you? Do you see benefit in applying this model to the patients I’ve just described?  Among those, what may be the most critical problems?  If you are given a free hand without constraint, what change do you hope to see the most at SKH? |

Thank you so much for participating today. You are welcome to contact us if there is anything in relation to the interview that you would like to talk about.

Before we end, can you recommend a colleague or two at working level who may also have insights about Generalist implementation in SKH whom we may talk to?

Thank you once again for your time today.

**Appendix B**

**Generalist (IGH) Implementation at Alexandra Campus**

| **Issue** | **Capability** | **Phase** |
| --- | --- | --- |
| A good proportion of patients with multiple conditions (especially the elderly) can benefit from more holistic care at the hospital. **Many of them may not require tertiary specialist care** | - One care team for each patient during inpatient stay, delivering holistic care - One principal doctor at outpatient clinic appointments, delivering holistic care | Phase 1 @ Alexandra Hospital |
| With the ageing population, the **volume of patients with multiple conditions is expected to grow** |  |  |
| **Transfers across care teams**, inpatient wards and care settings (e.g. RH-CH) risk fragmenting care further, especially for subsidised cases that require acuity transitions | - Minimal transfers during inpatient stay (from acute to rehab care), enabling coordinated safe care |  |
| **Transitional and community care** (especially those with more complex needs) can be facilitated further via targeted needs-focused interventions | - Patient segmentation (beyond medical needs), guiding appropriate interventions - One organised view of patient flow, enabling whole-of-person management - Systems-level care matching, providing timely and relevant choices | Phase 2 @ Queenstown |
| **Post discharge issues for access and care navigation** (e.g. lost to follow up) can be better addressed through improved information and care continuity |  |  |

**Appendix C**

The **Generalist (IGH) care model** integrates acute, sub-acute, rehabilitative, and community care, and is differentiated by the following elements:

**a. One Care Team during Inpatient Stay**

Holistic and coordinated care delivery is anchored by a lead physician who is supported by a multidisciplinary care team, reinforced through tertiary specialist support and anchored by five key clinical programmes.

Twice a week, a multi-disciplinary team of doctors, nurses, therapists, medical social workers, pharmacists and care managers meet up to look through each patients’ case notes and discuss the best consolidated care plan for the patient. In the IGH model, multi-disciplinary team rounds are not always doctor-led, but can be led by other members of the care team, depending on the patient’s needs at that point of time..

**b. Minimal Transfers during Inpatient Stay**

Resources and services provided by the care team are sensitive to the recovery needs of each patient. Acute care and rehabilitative care take place in the same ward, reducing the hassle and risks of handovers between disciplines, teams and institutions. The patient is also not transferred out to a community hospital but will be able to receive similar rehabilitative care at AH until he or she is fit for home.

The patient is cared for by the same care team from admission and treatment, through to rehabilitation and discharge. This provides the opportunity to build trusted relationships between patients, caregivers and the care team.

**c. One Principal Doctor at Outpatient Clinic Appointments**

There are five key programmes at AH designed to wrap care around the needs of patients. Under these programmes, multiple specialist outpatient clinic sessions are consolidated and helmed by one principal doctor in one appointment, thereby enabling holistic care of multiple chronic conditions, reducing visits and addressing issues like poly-pharmacy.

**d. Integration with the Community, Primary Care, and Home**

AH works with care teams in primary care and the community to facilitate seamless handovers and shared care. Discharge planning begins soon after a patient is admitted to the inpatient ward.

Close ties are being built and shared care processes worked out between AH and family physicians in the community, including named discharges of patients from AH to primary care, and direct access for patients to AH services through primary care referrals. To this end, CareHub@AH, a service helmed by care managers, expands the hospital’s current transitional care programmes.

CareHub@AH helps patients in the community navigate healthcare options, including hospital care and community care, facilitates management of medical and social emergencies, and collaborates with and taps on strong networks of community partners to better match resources to needs. For example, CareHub@AH provides care advice over the phone; efficiently contacts the individual’s primary physician; links patients to social support, day care, and home and community services beyond AH; and enables fasttrack access to AH services or fast track admission to hospital wards. During each admission, AH patients are introduced to the centralised hotline which is manned by care managers.

**e. Zero-based Design**

The IGH employs a zero-based design approach, where a clean slate is applied to designing, prototyping and innovating in areas like new care models, technology application and physical space.

**Appendix D**

Appendix D: Handout for Participants on Current SKH Care Model


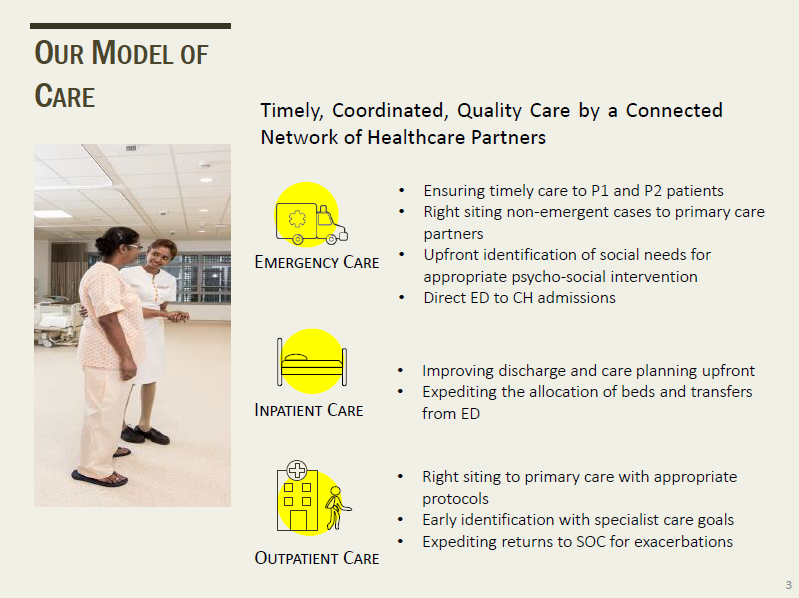


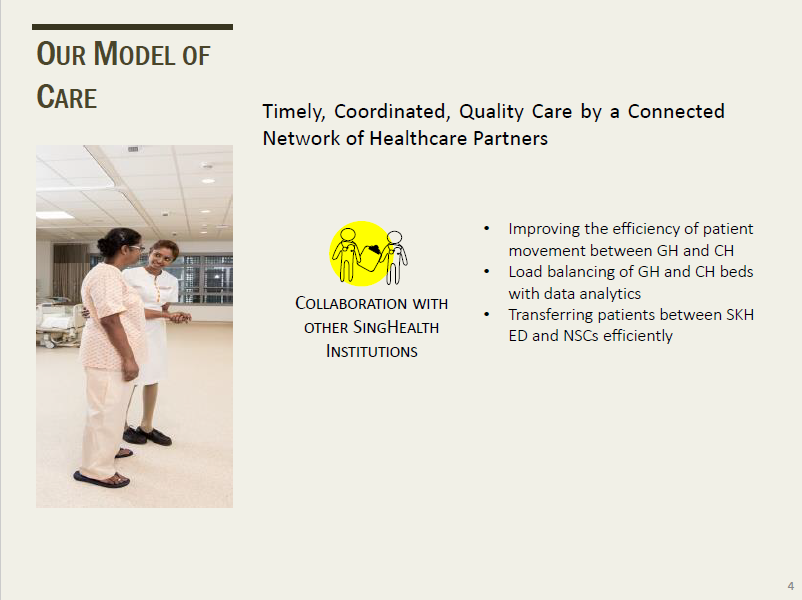


**Appendix E**


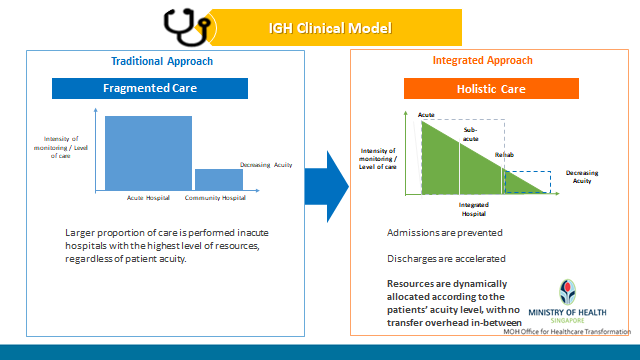


1. Porter, M., & Lee, T. (2013). The strategy that will fix health care. Harvard Business Review. Each IPU is organized around a medical condition with the full cycle of care coordinated across a multidisciplinary clinical team [↑](#footnote-ref-1)
